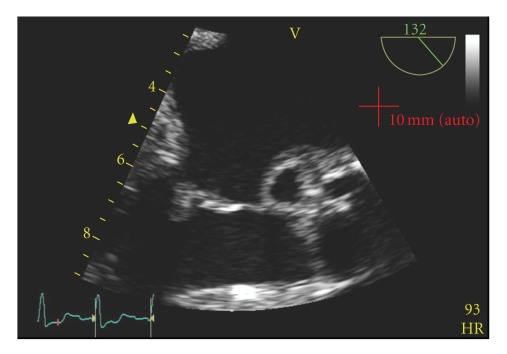# Prosthetic Valve Endocarditis: A Complication of Spinal Epidural Abscess

**DOI:** 10.1155/2010/105426

**Published:** 2010-12-20

**Authors:** Armando Bedoya, Bethany Gentilesco

**Affiliations:** ^1^Department of Medicine, Miriam Hospital, Providence, RI 02906, USA; ^2^The Warren Alpert Medical School, Brown University, Providence, RI 02912, USA

## Abstract

Epidural injections for chronic low back pain are controversial, and their effectiveness is debated. Although epidural injections are considered a minor procedure with low morbidity, catastrophic complications may occur. We describe a case of prosthetic valve endocarditis secondary to an epidural abscess after epidural injection to alert clinicians to this unusual association.

## 1. Case Report

**Figure 1:**
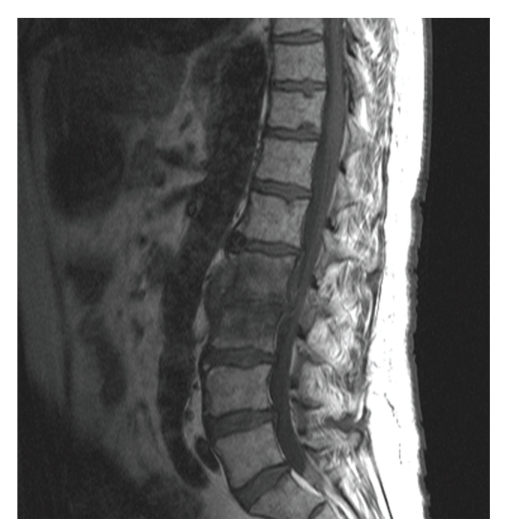


A 65-year-old man presented with fevers, shaking chills, generalized weakness, and back pain for one week. Past medical history significanly involved chronic low back pain and a porcine aortic valve replacement one year previously for symptomatic aortic stenosis. The patient reported that he recently had begun epidural steroid injections secondary to his failure to improve with conservative therapy, his last injection being one week prior to admission. His only medications included atenolol, aspirin, and irbersartan. He had no recent travels or procedures and denied intravenous drug usage. On exam, temperature is 102.4, heart rate was 84, and blood pressure 90/55 mmHg sitting with normal oxygen saturation. He was diaphoretic, having shaking chills. His exam was notable for a harsh systolic III/VI murmur best heard at left sternal border 2nd intercostal space with no significant neurological, ocular, pulmonary, abdominal, or skin/nail findings. Admission electrocardiogram showed normal sinus rhythm, left ventricular hypertrophy, and no ST or T wave changes. Chest X-ray was unremarkable. Laboratory results were significant for WBC = 18,900 with 4% bands and an erythrocyte sedimentation Rate of 56. MRI of the spine revealed osteomyelitis/discitis at L2-3 and a small epidural abscess at L2 (Figure 1). The abscess was too small to be aspirated by neurosurgery, and he was started on antibiotics. 

Given the presence of a new murmur with a prosthetic valve in the setting of an epidural abscess, endocarditis was suspected. Serial blood cultures grew coagulase negative staphylococcus. On hospital day number 2, a transthoracic echocardiogram showed no evidence of endocarditis. Due to continued high clinical suspicion of endocarditis, trans-esophageal echocardiogram on hospital day number 3 showed aortic valve vegetations with severe aortic valve regurgitation (Figure 2). The patient was continued on antibiotics. Since he was hemodynamically stable with no evidence of congestive heart failure, an emergent valve replacement was not indicated. His course was complicated by first-degree heart block on EKG, as well as acute tubular necrosis likely secondary to antibiotics. Blood cultures became negative after five days of treatment. He was discharged to complete a six-week course of antibiotics. He underwent an aortic valve replacement one month after his initial hospitalization. Surgery confirmed dehiscence of the valve as well as a subaortic root abscess that had eroded the aortic annulus. The site was debrided, reconstructed, and a new porcine valve was implanted. He was discharged on postoperative day six and has returned to work with no sequelae.

## 2. Discussion

This case illustrates an individual who failed conservative therapy for chronic low back pain and developed several complications from a common procedure. The temporal association of events suggests that the individual received his epidural injections for his chronic low back pain, which created a spinal epidural abscess (SEA), in turn seeding his prosthetic valve. A literature search found only six past case reports involving an infectious complication following a lumbar injection [1–6]. 

Epidural injections are currently an adjuvant therapy for chronic back pain. Although epidural injections are considered a minor procedure there are potential complications. The Wessex Epidural Steroids Trial reported headache as the most common side effect at 3.3% and nausea second at 1.7% [1]. A central nervous system infection, which complicated this case's treatment, from an epidural injection is a rare occurrence, but has a significant potential for morbidity and mortality. In one study, 6.3% of 128 community-acquired bacterial meningitis patients had a history of epidural injections [2].

An SEA is a rare occurrence but the incidence is rising especially with increase in spinal interventions. Currently 0.2–2 for every 10,000 patients present with an SEA, and several risk factors have been identified [3]. Intravenous drug users, skin infections, and abscesses all potentially cause transient bacteremia and subsequent hematogenous spread. Diabetes mellitus changes the integrity of the microvasculature allowing for a favorable environment for proliferation in the epidural space. Penetrating trauma and spinal manipulation, such as epidural injections, cause direct inoculation and potential contamination of the epidural space. However, in one third of the cases, no identifiable source is appreciated [4].

The route of infection can shed light on the potential organism. Skin infections and spinal manipulation account for the majority of cases, and gram-positive cocci, such as staphylococcus and streptococcus, account for more than half of the organisms cultured [5], with methicillin-resistant staphylococcus aureus cases steadily rising [6]. In addition to staphylococcus, intravenous drug users with an SEA can also be infected with pseudomonas. With immunosuppressed patients, mycobacterium, and fungi have also been isolated [5].

Clinically, the “classic triad” of pain, neurologic deficits, and fever has low sensitivity, and thus an assessment of risk factor for SEA has been advocated with particular focus on spinal manipulation, immunosuppression, and any potential source of transient bacteremia [7]. 85% of patients with SEA complain of back pain while only 35% complain of paresthesia, and the use of antipyretics makes the diagnosis difficult. On laboratory values, an elevated erythrocyte sedimentation rate has been found to be more sensitive and specific than an elevated white blood cell count and is suggested to be used as a screening test [7]. Currently, magnetic resonance imaging of the spine is the preferred modality for diagnosis [8]. Since neurologic recovery is directly correlated with the duration of the abscess, particular importance is placed on prompt diagnosis. Unfortunately, 75% of patients with SEA experience diagnostic delay (defined as multiple ED visits before diagnosis, admission without a diagnosis, or more than a 24-hour delay before diagnosis) [7]. Furthermore, despite adequate intervention the mortality rate still ranges from 6%–30% [5].

Spinal epidural abscesses have a pleomorphic, potentially misleading clinical spectrum and presentation. As illustrated in this case, fever in an individual with recent epidural injections should raise the suspicion of a spinal epidural abscess.

## Figures and Tables

**Figure 2:**